# Recent Progress in Manufacturing Techniques of Printed and Flexible Sensors: A Review

**DOI:** 10.3390/bios10120199

**Published:** 2020-12-03

**Authors:** Dinesh Maddipatla, Binu B. Narakathu, Massood Atashbar

**Affiliations:** Electrical and Computer Engineering Department, Western Michigan University, Kalamazoo, MI 49006, USA; binubaby.narakathu@wmich.edu (B.B.N.); massood.atashbar@wmich.edu (M.A.)

**Keywords:** additive manufacturing, inkjet printing, screen printing, gravure printing, flexography printing, flexible sensors, roll-to-roll manufacturing

## Abstract

This review provides an outlook on some of the significant research work done on printed and flexible sensors. Printed sensors fabricated on flexible platforms such as paper, plastic and textiles have been implemented for wearable applications in the biomedical, defense, food, and environmental industries. This review discusses the materials, characterization methods, and fabrication methods implemented for the development of the printed and flexible sensors. The applications, challenges faced and future opportunities for the printed and flexible sensors are also presented in this review.

## 1. Introduction

Sensors, which represent the “ears”, “noses”, or “eyes” for information processing systems, are being widely used in numerous industrial applications as well as in our day-to-day lives. Sensors are typically employed to provide real-time information, which has helped in advancing the electronic industry by simplifying modern technical systems and making many technical applications more cost effective, reliable, and safer. The need for sensing technologies has seen a dramatic increase in sensor R&D and applications over the past 25 years [1,2,3]. The remarkable advances in sensing technology, which have already been made possible and the potential range of applications that are yet to be developed, have placed sensors on the threshold of a revolution similar to that experienced by silicon technology in the computing industry during the 1980s [1,4]. An increasing demand for miniaturized, cost effective, and reliable sensors capable of monitoring multiple environmental, physical, chemical, and biological parameters thus requires the development of novel sensing devices with advanced capabilities.

Flexible hybrid electronics (FHE) is a rapidly emerging field, with significant commercial potential that drives investments in research and development within the electronic manufacturing industry [5,6]. FHE draws upon two primary threads: printed electronics (PE) and other advanced deposition/assembly processes, and semiconductor devices and packaging technologies [7,8]. FHE devices are a natural bridge between the silicon driven IC industry and the PE industry. Printing is an additive process that has expanded into electronic applications where electronic materials are deposited selectively using electrically functional inks in combination with standard printing processes [9,10,11,12,13,14,15]. This eliminates the need for masking and etching, thus resulting in a cleaner process that produces less waste than traditional methods. Due to the universal application of FHE technology, FHE markets are expected to experience exponential growth over the next decade with a global market for consumer and industrial products manufactured using this technology of up to $3 billion USD by 2030 [16].

The last few years have seen a noticeable increase in the development of wearable technology devices that are often worn on the human body [17,18,19,20]. These devices provide the user with information about various physiological parameters and surroundings. Currently, some of the markets for wearable devices are in sports, fitness, and healthcare [21,22]. Examples of these applications include Nike’s Fitbit exercise tracking and monitoring device and google contact lenses for diabetics that will provide a continuous readout on blood glucose levels [23,24,25,26]. The wearable tech market is forecasted to reach $302.3 million by 2023 [27]. More recently, with the advent of FHE devices, wearable devices that integrate novel flexible, stretchable or even tattoo-like sensors, consisting of stretchable electrodes and interconnects, which enable conformal and intimate skin-device contact, have been a major focus of several research groups and companies [17,20,28,29,30]. The development of these devices includes the deposition of functional materials on conformal and/or non-curvy surfaces in various designs.

The two main approaches in developing printed electronic devices are contact and non-contact printing [31]. In the contact printing approach, ink is transferred from the surface of patterned structures to the substrate by physical contact. Gravure, screen, and flexography printing are examples of contact printing processes [31,32]. In a non-contact process, the ink is transferred to the substrate via nozzles or openings, with no physical contact with the substrate. Inkjet and aerosol printing are frequently used for non-contact printing [31,32]. The emergence of FHE based on PE has started to increasingly revolutionize the field of sensing; both in industrial and consumer settings [33,34]. Flexible sensors can be used for curved surfaces, complex geometries, and foldable applications. In contrast, rigid sensors fabricated by conventional microelectronics techniques cannot provide the expected/desired responses on non-flat surfaces similar to the flexible sensors. In recent years, the efforts to gain a better understanding of the dynamics of printing flexible sensors has increased, and this interdisciplinary approach addresses the challenges of fabricating miniaturized, low-cost, flexible sensors via high-throughput techniques, which are expected to be used for applications in aerospace, automotive, environmental, packaging, biomedical, and defense [35,36,37,38,39]. PE enables fabrication of cost-effective sensors via large area printing due to roll-to-roll manufacturing capability utilizing solution-process techniques, large area substrates, multiple device printing per print batch, and fabricating in ambient conditions [36,37,38,39,40,41]. Modern fabrication technologies and the rapid maturation of application-based methodologies in PE have led to an increased understanding of semiconductor analyte interactions for use in chemical and biological detection. In addition, various types of physical sensors, such as strain sensors, optical sensors, temperature sensors, pressure sensors, and chemical sensors, including electrochemical sensors and gas sensors, have been realized using printing processes [42,43,44,45,46,47,48].

This review provides a detailed study on the dynamics of the PE by involving some common printing techniques, key materials and substrates, fabricated sensor types, challenges, and opportunities in the printed flexible sensors area. This review has the following structure: Section 2 discusses printable electronic materials and appropriate substrates, some important characterization methods to be considered for pre-print compatibility of materials and post-print quality analysis/assessments. The description of some commonly used non-contact and contact printing processes utilized for depositing solution-based materials and some examples of physical and chemical printed sensors will be discussed in Section 3. Finally, Section 4 brings together the technical challenges faced in the utilization of printing processes for the fabrication of flexible sensors and the opportunities enabled by the printing processes will be discussed.

## 2. Materials and Characterization Methods

### 2.1. Substrates

Printed electronics often require substrates that are uniform in smoothness, solvent resistance, stretchable, conformal, flexible, and light weight while providing both chemical and thermal stability. The substrates that are commonly used for fabricating FHE devices are poly (ethylene terephthalate) (PET), polyimide (PI), thermoplastic, polyethylene naphthalate (PEN), and thermoplastic polyurethane (TPU) [49,50,51,52]. As shown in Table 1, these substrates have different properties in terms of thickness, glass transition temperatures (T_g_), and transparency (for optical-based applications). In addition to these substrates, paper (specifically for fabricating disposable devices) and polydimethylsiloxane (PDMS) have also been employed for fabricating PE-based sensors and devices [49,52,53]. Each of these substrates is chosen for a particular application based on its properties. For example, PET excels in applications that require a very smooth surface of a few nanometers and optical transparency. PI substrates are widely employed for fabricating flexible PCBs due to their high glass transition temperatures and relatively higher mechanical as well as chemical strength, when compared to PET [54]. TPU and PDMS substrates are popular for developing stretchable devices, whereas as paper substrates are more common for developing cheap and disposable devices [55,56,57]. However, paper and TPU have a relatively higher surface roughness (≥ 1 µm) compared to other substrates [58].

### 2.2. Inks

The basic components of an ink system consist of functional elements, binders, solvents, and additives (Table 2) [59]. Typically, materials such as metals, semiconductors, and dielectrics are used as functional elements [59,60]. The most used metallic material, as a functional element, is silver (Ag) in the form of flakes, nanowires, and nanoparticles with a conductivity of ≈10^5^ S/m, which can be even improved using optimized annealing processes [61]. Carbon nanotubes (CNTs), carbon black (C), and graphene have also been used as functioning elements for different applications, including electrochemical sensing, humidity sensing, temperature sensing, and energy storage with a maximum conductivity of ≈10^2^–10^3^ S/m [62,63]. In recent years, research has been focused on using copper (Cu) (which is abundantly available and relatively cheap) as a functional element for metallic inks to replace Ag [64]. In addition to this, nickel (Ni), metallic composites (Ag/C), indium tin oxide (ITO), have also been used as conductive functional elements [65,66,67]. Semiconductors such as poly(3-hexylthiophene) (P_3_HT); titanium dioxide (TiO_2_); zinc oxide (ZnO); and dielectrics such as poly (methyl methacrylate) (PMMA), barium titanate (BaTiO_3_), polyvinyl pyrrolidone (PVP), polydimethylsiloxane (PDMS), and poly urethane (PU) have also been used as functional elements based on the application needs [59,65,68].

The other components of the ink system such as binders, solvents and additives, facilitate functional elements’ printability on flexible platforms [59,68]. Typically, binders are mixed with the functional elements in the presence of a compatible solvent, and they provide uniform film formation by crosslinking the functional elements after a curing process (thermal, UV or sintering) [59,68]. In addition to film formation, binders also provide the adhesion required for functional elements with the substrate along with gloss and resistance to humidity or ambient light conditions [59,68]. Cellulose-, alkyds-, rubber-, and acrylic-based resins have been used widely as binders. Water and a wide range of organic solvents including aromatic hydrocarbons, ethyl acetate, and alcohols (ethanol, isopropyl alcohol, etc.) have been used as solvents in the ink systems [59,68]. The solvents either uniformly dissolve or disperse the other components of the ink systems, facilitating the easy application of ink fluids on to the printing systems [68]. Recently, water-based inks have been gaining more attention due to low evaporation rates, low costs, and non-toxic nature [68,69]. Additives are used to modify/tweak certain ink systems’ properties in terms of wettability, surface tension, and pH [59,68]. For example, water-based inks have high surface tension, and surfactants as well as defoamers are used to reduce the surface tension and improve the wetting characteristics of the ink system [70]. In addition, humectants (hygroscopic material) are also used as additives to reduce the evaporation rates of the solvent in the ink system [68]. Multiple parameters must be considered when designing the ink system components in order to achieve a uniform ink film with desired characteristics without any coffee-ring effects and ink spreading that results in high raggedness.

### 2.3. Ink and Substrate Characterizations

The physical properties of the ink such as viscosity, surface tension, wetting characteristics, and density are crucial to consider for obtaining a better print quality [70,71,72,73]. These properties of the ink depend on the composition of the ink system components. Therefore, understanding these properties and measuring them as part of pre-print characterization can significantly impact the output quality of the printing processes.

#### 2.3.1. Rheometry

Rheology is the study of flow and deformation of fluid materials under applied forces (created due to stresses applied on it) [59,68]. It provides information on the behavior of the fluid systems under stresses, which is important in determining the compatible printing process, storage conditions (shelf life, anti-settling, and re-dispersibility), formulation (dispersion quality, stability, and viscosity adjustment) and quality of final film (thickness, smoothness, flow as well as levelling, and uniformity) [59,74,75,76]. Fluids can be typically classified as Newtonian and non-Newtonian fluids [77,78]. In Newtonian fluids, viscosity is constant over a wide range of shear rates and stress [77,78]. They have a single coefficient of viscosity for a specific temperature. On the contrary, non-Newtonian fluids cannot be defined by a single viscosity value, and they exhibit a variety of different correlations among viscosity, shear rate, and shear stress [77,78]. Typically, the ink fluid systems used for printed electronics are non-Newtonian fluids [79]. A device known as a rheometer is used for characterizing the rheological behavior of non-Newtonian fluids that require more parameters including shear stress, shear rate, and temperature to be set/varied to measure the corresponding variations in the viscosity values over time [76,80]. Typically, high viscosity inks ranging from 0.5 Pa.s. to 60 Pa.s. that exhibit thixotropic behavior are required for screen printing. Thixotropy is defined as the ability of the ink to exhibit relatively low/reduced viscosity, temporarily upon the application of shear, and then recover to its original/initial state (higher viscosity) when the shear is removed; thixotropic behavior facilitates the ink flow through the screen mesh resulting in ink transfer to the substrate and thus leads to ease of processability [66]. The gravure and flexo requires ink systems with viscosities ranging from 0.01 Pa.s. to 1.1 Pa.s. and 0.01 Pa.s. to 0.5 Pa.s., respectively, also typically thixotropic. In inkjet printing, for proper drop formation and jetting, viscosity of the ink should be below 0.1 Pa.s., which also may be thixotropic [31,66,81].

#### 2.3.2. Surface Tension

**Figure 1:**
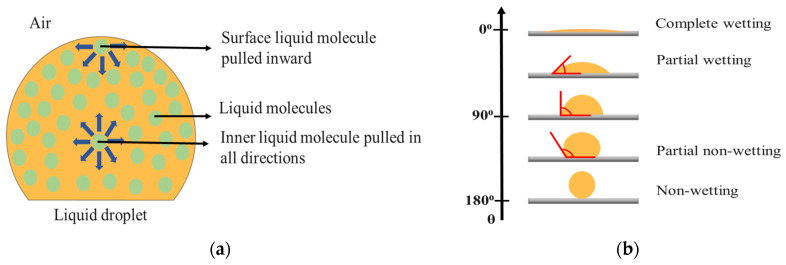
(**a**) Illustration of intermolecular attractive forces in a droplet and (**b**) wetting behavior of liquid drop-based on contact angle.

Surface tension is the property of a fluid’s interface and is defined as the elastic tendency of particles on the surface of the fluids resulting in the minimum possible surface area [82]. Surface tension is an important property that markedly influences many ecosystems. In other words, surface tension is described as the work required for disturbing the droplet shape of the fluid with intermolecular forces that holds the fluid together at any air or liquid interface, as shown in Figure 1a. It is work done per unit length, and the units are usually in dynes/cm or milli-newton/m (mN/m) [59]. The surface tension of any ink system depends on the solvents used in it and can be altered by adding a very small quantity of polar or non-polar surfactants to the ink system. There are many methods to measure the surface tension in the liquids, but the pendant drop method is typically used to obtain the surface tension which depends on analyzing the shape of the liquid droplet, in its biggest volume before it falls down on the surface of the substrate [59,83].

#### 2.3.3. Contact Angle and Surface Energy

The contact angle is often defined as the angle at which a liquid/vapor interface meets the solid surface (substrate) [84]. The contact angle is specific for any given system and is measured using a goniometer [59,66]. Contact angle measurement provides information about ink-surface interactions (the behavior of the ink droplet at the substrate surface) and its wetting behavior. For example, if an ink droplet does not spread well and forms a high contact angle (≥90°), this implies that the ink-surface interaction has poor wetting property (Figure 1b), and it is not desirable for printing applications. On the other hand, printing applications require good wetting property (contact angle < 90°). The contact angle depends on several factors such as surface roughness, ink formulation, solvent properties, surface energy, pre/post treatments (UV, plasma), and processing temperatures [59]. The goniometer helps to determine the contact angle and the spreading of the fluid on the surface of the substrate.

Surface energy is measured for solid materials (substrates) and is defined as the amount of intermolecular forces available at the surface of a material [85]. These forces can be exerted on to a fluid droplet to disrupt its interfacial bonds. Typically, surface energy of a substrate is measured using Owens and Wendt method (and new method: Altay–Ma–Fleming Method) and quantified in dynes/cm or mN/m [85]. Techniques such as corona, UV ozone, sintering, plasma, and laser treatments are employed to modify the surface energy of the substrates [59,86,87]. In printed electronics, the degree of wetting, spreading, and adhesion of a deposited ink mainly depends on the surface energy of the substrate and the surface tension of the liquid. In PE, it is always desirable to have the surface energy of the substrate at least above 7–10 dynes/cm compared to surface tension of the ink to get good wetting and adhesion characteristics [36,88].

#### 2.3.4. Surface Characterization Methods

Surface characterization is the study of the surface characteristics/properties of a solid material (e.g., substrates) [89]. In many applications, certain surface properties of the material such as roughness are crucial. These properties can easily be altered through interaction with the open environment due to physically or chemically adsorbed substances from gases, hydrocarbons, and water vapor [59]. Surface roughness has great influences on various other surface properties such as surface area, surface energy, electrical resistance, and thermal conductivity. Therefore, depending on the application needs, necessary modifications can be done to change roughness in order to achieve desirable changes in other surface properties. In PE, various applications require substrates with different surface properties. Before considering a material as a substrate, knowledge about its surface properties such as roughness, micro-mechanical properties, and thickness is of utmost importance to achieve a fine print quality. There are various surface characterization methods available to measure these parameters. Optical profiler and laser interferometry, scanning electron microscope, atomic force microscopy, Fourier transform infrared spectroscopy, X-ray fluorescence, and X-ray diffraction techniques are typically used to study the surface characteristics. In post-printing processes, many of these techniques are used to measure and analyze the structural properties, thickness, and roughness of the printed ink as well as print quality details such as raggedness and resolution.

## 3. Printing Processes and Applications

The printing processes are classified into impact and non-impact printing processes. In the impact printing process, the information on the printing plate/mask (information carrying medium) corresponding to image elements (allowing ink transfer) and non-image elements (no transfer of ink) is created on the substrate by the transfer of ink from plate to the surface of the substrate [90]. Screen, gravure, and flexography printing processes are classified as impact printing processes and require a printing plate or a fixed image carrier for information transfer to substrate. On the other hand, the non-impact printing (NIP) processes such as inkjet printing create/transfers information on to the substrate digitally and do not require any printing plate or physical information carrying medium [59,90,91]. Even though the NIP processes seem attractive compared to impact printing processes, each printing method has its own advantages and provides a specific printed feature size.

The widely used printing techniques that are employed for the development of printed electronics are screen, inkjet, flexographic, and gravure printing. Each printing process requires a particular set of ink properties and results in specific printed features as shown in Table 3 [59,68]. Screen printing requires very high viscosity inks with high functional element solid content and has been used for depositing thick film layers with high repeatability. On the other hand, inkjet printing requires very low viscosity inks and is being used for obtaining thin film layers with high resolution. Similarly, flexography and gravure printing require medium viscosity inks and are completely compatible for roll-to-roll (R2R) high volume production. For example, if the desired thickness of the printed film for a particular application is 20 µm and the viscosity of the available ink system is 30 Pa.s, then the screen printing process can be employed.

### 3.1. Screen Printing

**Figure 2:**
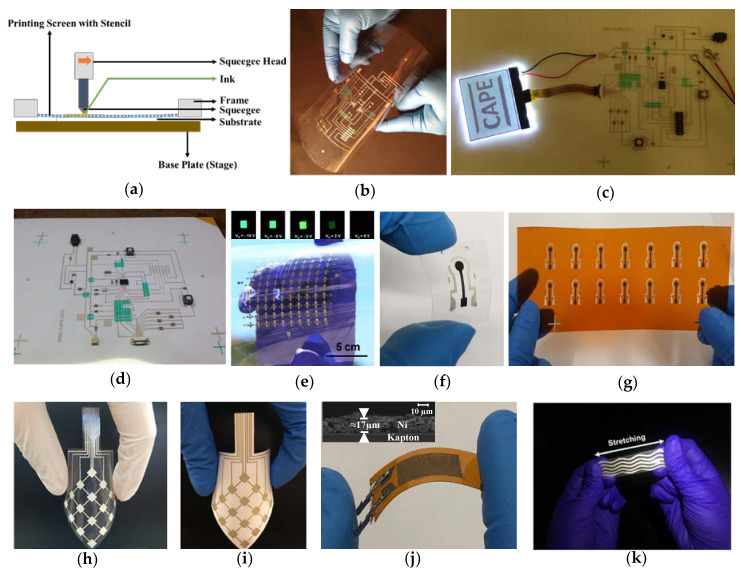
(**a**) Schematic of screen printing process; screen printed multilayered flexible circuits on (**b**) poly (ethylene terephthalate) (PET), (**c**) glass, (**d**) paper platforms [93], © IEEE, Reprinted with permission from IEEE Transactions on Components, Packaging and Manufacturing Technology; (**e**) screen printed top-gated TFTs, Reprinted with permission from [94], © American Chemical Society; (**f**,**g**) flexible and planar electrochemical sensors on PET and PI substrates (2 × 1 cm^2^) [98,99], Reprinted with permission from RSC and IEEE Sensors Journal; (**h**), and (**i**) polyvinylidene fluoride (PVDF)-based touch sensor on PET and paper platforms, Reprinted from [100] with permission from Elsevier; (**j**) Ni-based resistance temperature detector (RTD) on polyimide substrate (2.5 × 1.2 cm^2^) [101], © IEEE, Reprinted with permission from IEEE Access; (**k**) flexible and stretchable strain sensors, Reprinted from [102] with permission from Elsevier.

Screen printing is a technique in which a paste-like material (ink) is transferred onto the substrate. Screen printing is typically done either by hand or using a semi or fully automated system. The screen printer consists of a squeegee, stencil, and a screen with the design on it (Figure 2a) [59,90]. Typically, screen fibers are made of plastic, natural silk, or metal fibers, and the squeeze is made of rubber. A section in the screen will be imposed by the design of the desired print and the ink is allowed to pass through it, and the desired design is created on the substrate being used. The ink is transferred either by pushing or forcing the ink by squeezing through the screen, and thus, this printing process is also referred as push through process. The print quality mainly depends on the mesh count, wire diameter, emulsion thickness, off-set height, and deflection angle of the screen. The screen-printing technique has the capability to print electronic devices at a low cost, with very little or no material wastage and provides an end product that can be extremely flexible [91,92]. With screen printing capabilities including R2R manufacturing and processing at ambient conditions, it is possible to fabricate a large number of printed devices in a relatively short period of time.

There are many flexible circuits, electronic devices, and sensors fabricated using screen printing process, and their functionality has been demonstrated with performance comparable to traditional/conventional devices. For example, Eshkeiti et al. fabricated prototypes of multilayered printed circuit boards (PCBs) prototypes with Ag and UV acrylic inks as metallization and dielectric layers, respectively using AMI 485 semi-automatic screen-printing press [93]. They were the first research group that implemented fully operational three-layered flexible PCB prototypes on PET (Figure 2b), glass (Figure 2c) and paper (Figure 2d) substrates with electronic components and microcontroller populated using pick and place equipment to drive a liquid-crystal display of 160 × 100 pixels. The resistance of the printed lines increased by only ≈1.8%, after subjecting to 10,000 cycles of bending indicating the robustness of the printed and flexible PCB prototype. Cao et al. reported the first fully fabricated screen printed top-gated TFTs using semiconductor-enriched SWCNT as channel materials, Ag as source, drain as well as gate, and high-k barium titanate as dielectric layer on PET substrate [94]. The TFTs had a mobility up to 7.67 cm^2^ V^−1^ s^−1^ with low operating voltage of less than 10 V and current on/off ratio between 10^4^–10^5^ with superior mechanical flexibility. They demonstrated the capability of the printed TFTs by controlling the intensity of external OLEDs shown in the inset of Figure 2e. Three electrode configurations on PET and polyimide were screen printed with Ag, C, and Ag/AgCl as counter, working and reference electrodes, respectively, by Dr. Atashbar’s research group, to selectively detect various heavy metals including mercury, lead, cadmium, and copper in drinking water (Figure 2f,g) [95,96,97,98]. These flexible and planar electrochemical sensors are compatible with all types of traditional electrochemical techniques including cyclic voltammetry, amperometry, and differential pulse voltammetry, and has detection capability well below the toxicity levels set by the U.S. environmental protection agency (EPA) and world health organization (WHO). In addition, the printed electrochemical sensor presented by Maddipatla et al. provided lower limit of detection (LOD) and limit of quantification (LOQ) compared to colorimetric and optochemical sensors [98,99].

Emamian et al. successfully fabricated a complex piezoelectric-based touch sensor using screen printing process [100]. The sensor consists of a polyvinylidene fluoride (PVDF)-based piezoelectric layer sandwiched between the top and bottom Ag electrode layers. All these layers were completely screen printed on PET (Figure 2h) and paper (Figure 2i) substrates, and the polarization of the PVDF layer was investigated by performing capacitance-voltage analysis. The PET-based touch sensor has a sensitivity of 1.2 V/N whereas paper-based sensor exhibited 0.3 V/N demonstrating that these sensors have the potential to be employed as touch sensors or energy harvesters in robotics and automotive applications. Various temperature, strain, and pressure sensors have also been realized using additive screen-printing process. Turkani et al. reported nickel (Ni)-based resistance temperature detector (RTD) on polyimide substrate for detecting wide range of temperatures varying from −60 °C to 180 °C (Figure 2j) [101]. The flexible RTD exhibited a relative resistance change of 113% with a temperature coefficient resistance (TCR) of 0.44%/°C and response time of <10 s. The thin RTD film was also very stable and repeatable across the wide temperature ranges. Yoon et al. and Bose et al. fabricated flexible and stretchable strain sensors using screen printing process by depositing Ag ink on TPU substrates (Figure 2k) [102,103]. Multiple configurations including wavy, meander lines, and horseshoe-type were used to detect the applied strains [102,103,104,105]. A 20% strain was detected by the strain sensor with wavy configuration and demonstrated excellent stretchability when compared to conventional strain sensors. These multiple research works demonstrate the feasibility of employing screen-printing process for the development of various cost-efficient and high-performance electronic devices, sensors, and circuits [106,107,108,109]. Currently, screen printing is one of the most employed processes in the production lines of printed electronics.

### 3.2. Inkjet Printing

**Figure 3:**
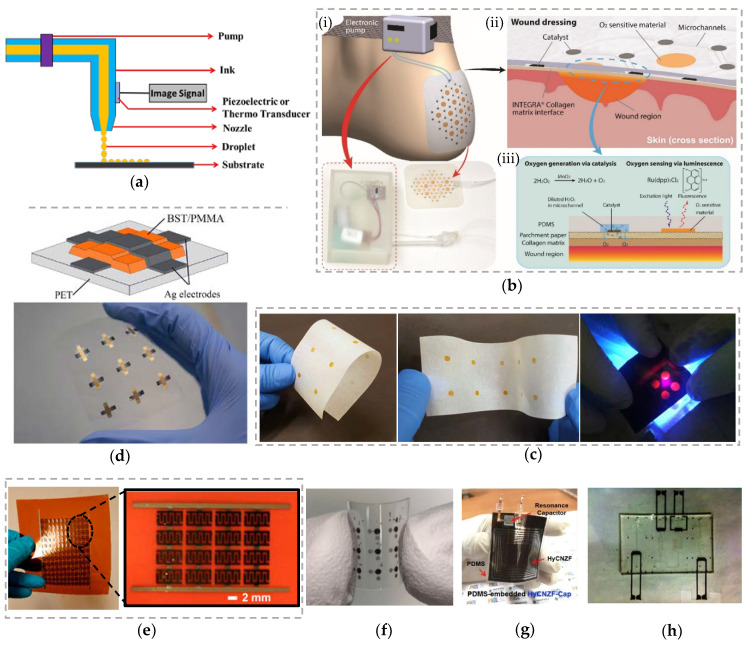
(**a**) Schematic of inkjet printing process; (**b**) flexible paper-based bandage with continuous oxygen delivery and sensing capabilities developed to treat the chronic wounds: (i) overview illustration of the patch in use for foot ulcer applications, (ii) cross-sectional view of oxygen generation and sensing patch and wound area, (iii) mechanisms for generating and sensing oxygen on a flexible smart wound dressing [70]; (**c**) paper-based oxygen sensors [114], Reprinted with permission from RSC; (**d**) fully inkjet-printed metal–insulator–metal capacitors [116]; (**e**) graphene-based micro-supercapacitors using electrochemically exfoliated graphene as electrodes and current collectors, Reprinted with permission from [117], © American Chemical Society; (**f**) multilayered flexible organic Schottky diode on PET substrate [118], © IOP Publishing, Reproduced with permission from IOP; (**g**) flexible wireless power transfer module (42 × 42 mm^2^), Reprinted from [119] with permission from Elsevier; (**h**) microfluidic-based sensing platforms with inkjet-printed Ag electrodes (3.8″ × 2.5″ × 0.2″) [120], © IEEE, Reprinted with permission from IEEE Sensors Journal.

Inkjet printing is a non-impact printing method (NIP), and it uses a digital image signal to print the design by propelling ink droplets onto the substrate instead of any physical image carriers [59,90]. Typically, in inkjet printing, the inks will be in a liquid state with low viscosities and can be directly printed onto the substrate. Inkjet printing is classified into continuous inkjet printing and drop-on-demand (DOD) inkjet printing. In continuous Inkjet printing, a continuous flow of ink droplets is controlled electronically by a voltage source. Some of the ink droplets are subjected to electrostatic charge and are deflected to get a negative print by electrostatic deflectors while the uncharged drops are used to print the desired image onto the substrate. Thermal inkjet printers are very popular in graphic printing and packaging industries. In a DOD-based inkjet printing, the ink droplets are created only if it is required by the image signal to get the desired print [59,90]. The droplets are formed either by using thermal or piezoelectric techniques as shown in Figure 3a. In thermal inkjet printers, the ink droplets are forced or impelled out of the cartridge nozzle by a vapor bubble formed because of the vaporization of ink liquid. In piezoelectric inkjet printers, the volume of the cartridge nozzle is mechanically deformed based on the image signal and the ink droplets are released from the nozzle. DOD-based inkjet printing is very popular in FHE and has several advantages including as mask-less fabrication, high print resolution, cost efficiency, and scalability from table-top devices to big press units [90,110,111,112].

Various devices have been fabricated using inkjet printing process. Ochoa et al. developed flexible paper-based continuous oxygen delivery and sensing bandage platform using inkjet printing to treat chronic wounds (Figure 3b) [70,113]. The authors employed parchment paper as the base platform of the dressing and inkjet-printed manganese oxide (MnO_2_) and ruthenium-based inks on parchment paper for locally generating and measuring oxygen in a wound region. By varying the density of the inkjet-printed MnO_2_ deposited, the generation of the oxygen concentration was controlled. The fluorescence property of inkjet-printed ruthenium on the paper substrate facilitates contact less measurement of oxygen at a wound site (Figure 3c) [114]. This multi-functional smart wound healing bandage is designed as a wound dressing platform that offers various properties of wound dressings (e.g., mechanical strength and flexibility) while featuring additional ones not found in conventional wound dressings (e.g., on-site generation of oxygen, delivery of oxygen or other therapeutics, and integration of sensors on the same substrate). Typically, wounds vary from one to the other, and even the extent of tissue damage across any single wound site is not uniform and requires different concentrations of oxygen and other therapeutics across the wound site. Implementation of inkjet printing is very pivotal for this research due to its rapid, mask-less customization of designs for accelerated dressing development as well as for mass customization. In other words, inkjet printing facilitates rapid fabrication of customized wound bandages in terms of dimensions/size as well as the intensity of the oxygen and other therapeutics to match the specific requirements (provided by the clinicians) of an individual wound. The significance of this research is based on the contribution that this project is bound to have towards the field of wound treatments [115].

Mikolajek et al. prepared fully inkjet-printed metal–insulator–metal capacitors (Figure 3d) [116]. They deposited Ag as metal electrodes and BST/PMMA as insulator or dielectric material on PET substrate using inkjet printing process. This printing process deposited thin, very uniform, and smooth layers with high resolution and relatively less pinholes. Therefore, the printed BST/PMMA composite-based dielectric layer exhibited 7 to 18 times higher dielectric constant when compared to pure PMMA. In addition, Li et al. fabricated graphene-based micro-supercapacitors using electrochemically exfoliated graphene as electrodes and current collectors, and polyelectrolyte ink (made using poly(4-styrenesulfonic acid) as a solid-state electrolyte (Figure 3e) [117]. The micro-supercapacitors have an aerial capacitance of 0.7 mF/cm^2^, and when connected in an array of over 100 devices on a flexible polyimide substrate, the devices can be charged to 12 V. The super capacitors were able to retain their performance over 8 months even without any encapsulation due to the use of polyelectrolyte ink. Further, Cao et al. developed a multi-layered flexible organic Schottky diode on flexible PET substrate using inkjet printing process (Figure 3f) [118]. Ag ink and poly(3,4-ethylenedioxythiophene) polystyrene sulfonate (PEDOT:PSS) was used as bottom and top electrodes, respectively. A poly(3-hexylthiophene (P3HT) was employed as a semiconducting layer sandwiched between the top and bottom electrodes. A precise control on fine ink deposition and better ink flow which are very crucial for multilayer devices was achieved by using inkjet printing and including microfluidic networks with capillary channels and flow stoppers in the diode fabrication. The printed diodes exhibited a high rectification ratio of 5 × 10^4^ with negligible hysteresis and high durability in bending tests.

Bissannagari et al. developed a flexible wireless power transfer module using inkjet printing process (Figure 3g) [119]. In this work, a 3D nickel (Ni)-Zinc (Zn)-ferrite (NZF)-based trench structure hybridized with alternative layers of Ag and PI in a spiral pattern was created using inkjet printing to fabricate a flexible power receiving coil. In addition, a resonance capacitor with Ag as top and bottom electrodes; BaTiO_3_ and PI infiltrated BaTiO_3_ as dielectric layers was also fabricated using inkjet printing process and integrated to power receiving coil in order to fine tune the resonance frequency of the coil to 6.78 MHz. The coil with capacitor was embedded in to PDMS films using casting method to serve as encapsulant as well as to reduce the bending/physical stress on the coil. This wireless module was able to successfully charge a mobile phone or a smart watch even at a distance of 40 mm. Narakathu et al. developed flexible microfluidic-based sensing platforms in which the Ag-based electrodes were inkjet-printed to detect and quantify various concentrations (as low as picomolar levels) of toxic chemicals such as mercury sulfide and cadmium sulfide using impedance spectroscopy (Figure 3h) [120]. Due to the attractive features such as mask-less fabrication and high print resolution, various other devices such as SERS substrates for the detection of heavy metals, gas sensors, humidity sensors, thermistors, and antennas were also realized using inkjet printing process [121,122,123,124,125,126].

### 3.3. Gravure Printing

**Figure 4:**
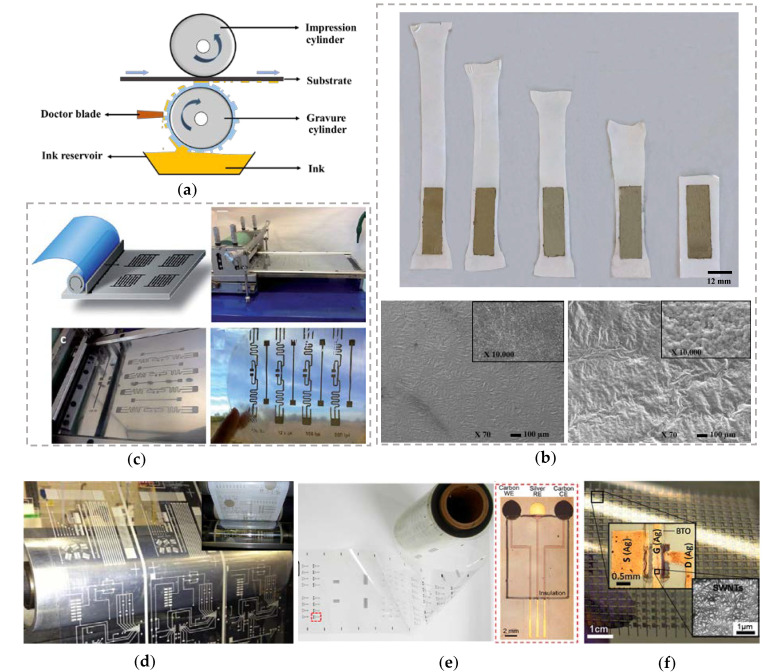
(**a**) Schematic of gravure printing process; (**b**) wrinkle-structure-based SERS substrate with SEM micrographs showing the plain and wrinkle structures on stretchable thermoplastic polyurethane (TPU) substrate, Reprinted from [131] with permission from Elsevier; (**c**) stable transparent nanopaper-based RFID antenna [132], Republished with Permission of RSC Pub; (**d**) R2R manufacturing of large area printed circuits and antenna structures; (**e**) electrochemical sensors on a PET roll for detecting ions, metabolites, heavy metals, and other small molecules, Reprinted with permission from [136], © American Chemical Society; (**f**) fully printed CNT-based high performance TFT arrays, Reprinted with permission from [137], © American Chemical Society.

Gravure printing is an impact-based printing technique that provides economical production with fast outputs along with high-quality printing. It uses low viscosity inks and is known for its robustness. The image carrier cylinder (gravure cylinder), doctor blade, ink reservoir, and the impression cylinder are some of the main components of the gravure printer as shown in Figure 4a [59,90]. Typically, the impression cylinder is made of rubber, the gravure cylinder is made of copper coated steel, and the doctor blade is manufactured with steel. In gravure printing, the image of the desired design is engraved on the surface of the gravure cylinder. The entire gravure cylinder is flooded by ink from the ink reservoir, and the extra ink is wiped off from the cylinder with the doctor blade prior to printing. The ink is transferred onto the substrate from the gravure cylinder with high pressures, and the substrate movement is controlled by the impression cylinder.

Antennas, SERS substrates, TFTs, electrochemical sensors, pressure sensors and many more devices has been realized using gravure printing process [127,128,129,130]. For example, Maddipatla et al. fabricated wrinkle-structure-based SERS substrate for the detection of illicit drugs such as cocaine (Figure 4b) [131]. The wrinkle structures were created by subjecting the TPU substrate under varying strains (25%, 50%, 75%, and 100%) and printing Ag ink with 150 nm particle size on stretched TPU substrate using gravure printing process. Enhanced Raman signal intensity of cocaine (enhancement factor of 6) was obtained due to the generation of larger electromagnetic fields. This was attributed to the increase in the number and depth of hotspots caused by wrinkle patterned structures. In addition, Zhu et al. fabricated for the first time a paper-based RFID antenna using gravure printing process (Figure 4c) [132]. In this work, they prepared a stable transparent nanopaper by treating nanocellulose fiber with glutaraldehyde treatment and hydrochloric (HCl) and UHF RFID antenna tag “squiggle” was developed by depositing water-based Ag ink on the nanopaper using gravure printing process. Insertion losses of −37.9 and −38.85 dB were obtained at the maximum gain of 683.75 MHz for the 100 lpi and 120 lpi printed antennas, respectively.

Dr. Atashbar and Dr. Joyce research groups from Western Michigan University were the first to demonstrate large scale R2R fabrication of printed circuits and antenna structures using gravure printing on both PET and paper substrates using conductive Ag ink in 2012 (Figure 4d) [133,134,135]. Further, Bariya et al. fabricated various electrochemical sensors on a PET roll for detecting ions, metabolites, heavy metals, and other small molecules (Figure 4e) [136]. The sensors comprised of carbon as working and counter electrode, Ag as reference electrode, and polyethylene resin as encapsulant. To demonstrate the functionality of the printed electrodes, various sensing layers that can selectively detect pH, sodium, potassium, glucose, and caffeine were deposited on the printed arrays. Lau et al. developed fully printed CNT-based high performance TFT arrays using gravure printing process (Figure 4f) [137]. Ag nanoparticle-based ink was gravure printed as source, drain, and gate electrodes on SWCNT coated PET substrate. Then the insulator was also gravure printed using barium titanate nanoparticles. The top gated fully oriented TFT exhibited excellent performance with a mobility of ~9 cm^2^/(V s), on/off current ratio of 10^5^ and features minimal hysteresis, high flexibility, and operational stability.

### 3.4. Flexographic Printing

**Figure 5:**
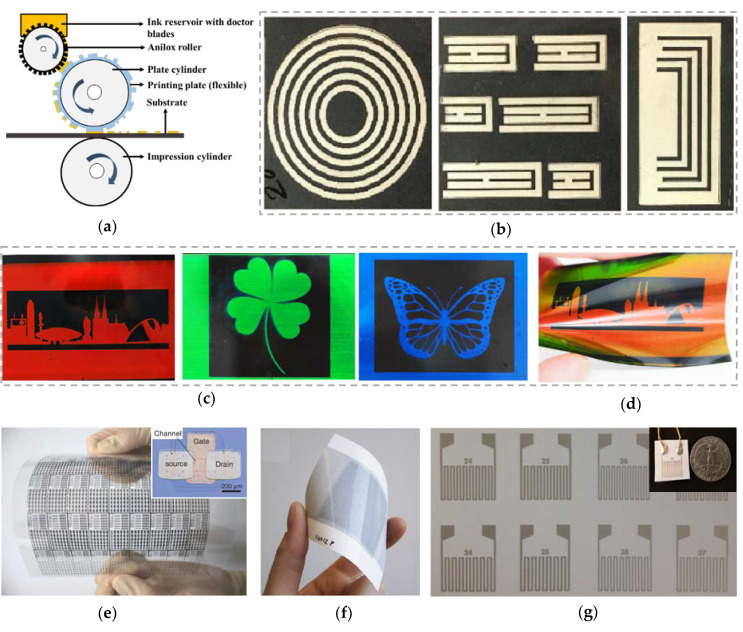
(**a**) Schematic of flexography printing process; (**b**) flexible chip less RFID tags (5-bit ring resonator tag (34 × 34 mm^2^), 3-bit UH-strip tag (30 × 30 mm^2^) and 4-bit U-slot tag (34 × 16 mm^2^)) [138], © IOP Publishing, Reproduced with permission from IOP; (**c**)and (**d**) chiral nematic oxetane liquid crystals (LC) on PET platform (3 × 3 cm^2^), Reprinted with permission from [143], © American Chemical Society; (**e**) high mobility flexible CNT-based TFTs on PEN substrate (15 × 15 cm^2^) [144], © (2013) The Japan Society of Applied Physics; (**f**) fully printed flexible large area loud speaker on a paper platform (16 cm^2^), Reprinted from [145] with permission from Elsevier; (**g**) Ag-based strain sensors on paper platform [146], © IEEE, Reprinted with permission from IEEE Sensors Conference Proceedings.

Flexographic printing is a well-established R2R high throughput rotational printing method. It is an indirect impact-based printing technique that can provide a wide range of ink thicknesses with the same resolution. The main parts of the flexographic printer are anilox roller, an impression cylinder, doctor blade, and the ink reservoir (Figure 5a) [59,90]. Anilox roller (a steel cylinder), which has finely engraved cells on its surface made of chromium or ceramics, collects the specific amount of ink from the ink reservoir. Then, the collected ink is transferred onto the elevated structures of the printing plate. With the help of the plate cylinder, the ink on the printing plate is transferred on to the substrate. It is capable of addressing problems such as contact finger geometry as well as production line output. In addition, flexography has high speed R2R printing capability (up to 100 m per minute) when compared to inkjet and screen printing [138,139]. Flexography printing is a well matured printing technique in graphic printing and packaging industries. However, the implementation of flexographic printing in FHE is not yet explored enough due to the limitations in research and commercial availability of functional inks compatible to flexographic printing. Therefore, there is only limited literature available on the development of flexography-based FHE devices [140,141,142].

Shrestha et al. developed flexible chipless RFID tags using a benchtop flexography printer (Figure 5b) [138]. Three resonators (3-bit UH-strip tag (30 × 30 mm^2^), 4-bit U-slot tag (34 × 16 mm^2^), and 5-bit ring resonator tag (34 × 34 mm^2^)) were fabricated by depositing water-based Ag ink on three different substrates (PET, thermal paper, and PVC). The printed tags were evaluated for their microwave performance, and the electromagnetic responses were used to obtain the tag IDs. The radar cross-section amplitudes of the tags printed on PET demonstrated relatively better performance when compared to the tags printed on other substrates. The three printed tags generated pre-defined resonances between 1–10 GHz in the unlicensed ultra-wide band frequency spectrum. Hoekstra et al. developed novel photonic materials based on chiral nematic oxetane liquid crystals (LC) using flexographic printing process for potential application as anticounterfeit labels (Figure 5c) [143]. To create the LCs, different concentrations of oxetane-based materials were mixed with cationic photo initiators and deposited on the biaxially oriented 36 µm PET substrate using flexographic printer. Then, the printed patterns were thermally cured to align the LCs and photopolymerized using a UV dryer. As shown in Figure 5d, the patterns consist of both chiral nematic and isotropic regions with high resolution.

Higuchi et al. developed high mobility flexible CNT-based TFTs (Figure 5e) [144]. In this work, they deposited Ag nanoparticle ink, polyimide ink and resist ink with Nmethyl-2-pyrrolidone (NMP) solvent as electrodes (source, drain, and gate), gate insulator and CNT patterner, respectively on to a plasma treated PEN film using flexographic printing process. The CNTs grown by chemical vapor deposition technique was transferred on to TFT electrodes. The printed TFT exhibited a high mobility of 157 cm^2^/Vs with an ON/OFF ratio of 10^4^. Hubler et al. reported the first fully printed flexible large area loudspeaker on a paper platform. PEDOT:PSS and poly(vinylidene fluoride-trifluorethylene (P(VDF-TrFE)) was deposited as electrodes (top and bottom) and piezoelectric layer, respectively, on a matt coated wood-containing paper using flexographic printing (Figure 5f) [145]. The P(VDF-TrFE) layer was sandwiched between the electrodes, like a capacitor. The samples were polarized by contact-poling process across an effective area of 16 cm^2^. The speakers were able to generate 80 dB sound and exhibited stable performance over six months in air without any encapsulation. In addition, Maddipatla et al. developed a paper-based strain sensor using flexography printing (Figure 5g) [146]. The authors printed Ag-based strain gauges with different meander lengths using pilot scale flexography press at 30 FPM. The printed flexible strain sensors, subjected to 3-point bend tests, were able to detect minute displacements as low as 1 mm with repeatable performance over 500 cycles.

## 4. Conclusions and Outlook

The development of FHE-based devices using various additive printing processes is growing exponentially due to obvious reasons—rapid-large area fabrication, cost efficiency, thin and light weight devices. In this paper, the significance of printed electronics and the parameters to be considered prior to printing and to be measured post-printing along with the numerous prototype devices developed using each printing process is presented. There are still a few more scientific challenges that need to be addressed to effectively perform research and adopt printing technologies for developing FHE-based devices. For example, the screen-printing process provides high wet film thickness when compared to other printing processes resulting in high spreading of ink and low resolution if not cured instantly [59]. In addition, the ink will be exposed to atmosphere for long times while printing, leading to solvent evaporation and deterioration of the mesh quality [59,147,148]. In inkjet printing, developing inks for proper jetting of droplets in a specified area and cleaning cycles is challenging due to the rate of evaporation of solvents and agglomeration of active particles, which leads to clogging of nozzles [149,150]. In addition, the one-time use cartridges are expensive and, due to the limited number of nozzles and slow speed of inkjet printing process (to avoid the deflections in the droplets during the time of flight), the throughput/yield rate is relatively low when compared to other printing processes [149,150].

In gravure printing, the gravure cylinder with pattern engravings is relatively expensive, and during the printing process, a tiny portion of the ink gets clogged or remains in the cells (etched walls) of the cylinder and dries out impacting the quality of the subsequent prints [151]. In both flexography and gravure, the availability of functional inks is very low. Both of the printing processes are well established in graphic printing industries and meant for continuous large-scale production. To develop inks compatible for these two processes requires large volumes of functional materials during the research phase with associated high costs. Due to these reasons, the research activities for these two processes are still less when compared to screen and inkjet (requires a lower quantity of inks). In addition, the outer edges of printed patterns in flexography typically suffer from squashed-ink appearance due to numerous reasons (insufficient ink volumes, inappropriate anilox roller volume, ink drying, viscosity, speed of machine) [59,152].

Due to these unresolved issues in printing, there are huge variations in the fabrication of FHE devices (both device-to-device and batch-to-batch) leading to unreliable and unstable performance. In addition, the variations in the environmental conditions including temperature and humidity degrades the electronic performance of FHE devices. To overcome these major challenges that FHE currently faces, the standardization of various parameters that could provide devices with high reliability, repeatability, and robustness is required. In order to establish standards and mature the FHE research area, a data cube must be developed with all the parameters that can impact the electronic performance of each FHE device including simulation tools (to design, model, and simulate the complete device), pre-print characterizations (viscosity, shear rate, surface tension, wetting properties of ink, surface roughness, thickness, gas permeability, and defects in identification of substrate), printing process parameters (deposition conditions of the printer, ambient temperature, and humidity), post-print characterizations (print film thickness, roughness, coverage, raggedness, and functionality), mechanical tests (bending, twisting, and flexing), and shelf-life characterizations (lifetime of each material and the device). Around the globe, many manufacturing innovation institutes (NextFlex, San Jose, CA, USA; HI-RESPONSE, Swindon, UK, and MADRAS consortiums, Barcelona, Spain, Europe) are working in collaboration with numerous scientists and experts in both academia and industry to address the challenges associated with the printing processes and set the standards to advance the manufacturing ecosystems of the rapidly expanding field of FHE [153,154,155,156]. Adopting these novel additive printing technologies on flexible platforms would potentially lead to cost-efficient and flexible devices [157,158,159,160] and is being envisioned to revolutionize diverse applications in many fields including food, agriculture, defense, automotive, and biomedical in the next few years.

## Figures and Tables

**Table 1:** Specifications of various substrates employed in printed electronics (PE).

Substrate	PET	PI	PEN	TPU	Paper	PDMS
**Classification**	Thermoplastic Polymer				Cellulose Fibers	Silicone Elastomer
**Thickness (µm)**	13–356	25–125	12.5–250	25–500	20–250	125–4775
**Roughness (µm)**	≈0.04	≈0.07		≈0.025	0.02–5	~0.24
**Tg (°C)**	81–150	360–410	120–200		~80	145–150
**Density (g/cc)**	1.38	1.42	1.33	1.32	0.6–1.0	0.97
**Young’s Modulus (GPa)**	2–3.2	2.76	2.2–3.0	2.41	0.5–3.5	0.57–3.7
**Folding Endurance (Cycles)**	>800	5000–285,000	>1000	2,000,000		-
**Comments (Pros, Cons, Applications)**	*Pros*: Transparency, Smoothness, Economical Compared to Other Thermoplastic Polymers	*Pros*: High Thermal, Mechanical and Chemical Resistance	*Pros*: Transparency, Relatively Higher Thermal and Mechanical Stability than PET	*Pros*: Stretchable Unlike Other Plastic Polymers, Mechanical and Chemical Resistance, Less Gas Permeability than PET	*Pros*: Cheap/Low Cost, Ecofriendly, Available in Abundance	*Pros*: Biodegradable, Non-Toxic, High Stretchability
	*Cons*: Low Tg	*Cons*: Non-Transparent, Relatively High Cost	*Cons*: High Cost than PET, Not as Good as PI in Terms of all Properties Except Cost	*Cons*: Relatively High Cost	*Cons*: Low Mechanical and Chemical Resistance	*Cons*: Low Mechanical and Chemical Resistance, Hydrophobic Surface (Low Surface Energy)
	*Applications*: Optical, Simple Printed Circuitry, Occupant/Pressure Sensors	*Applications*: Electrochemical Sensing, TFTs, Flexible PCBs	*Applications*: Flexible Heaters, Opto-Electronic Sensors, Highly Used in Packaging as Labels and Laminates, Printed Circuits, and Optical Displays	*Applications*: Wearable Electronics	*Applications*: Disposable Devices and Sensors	*Applications*: Wearable and Biomedical Devices Including Microfluidics and Sensors

**Table 2:** Different components present in the ink system.

Ink System Components (wt. %)				
Printing Process	Functional Element	Binder	Solvent	Additives
Screen	35–55	30–20	30–20	1–5
Inkjet	5–10	5–20	65–95	1–5
Flexo	12–17	40–45	25–45	1–5
Gravure	12–17	20–35	60–65	1–2

**Table 3:** Specifications of printing techniques employed in printed electronics.

Printing Process	Image Carrier	Film Thickness (µm)	Printing Speed (m/min)	Resolution (µm)	Viscosity (Pa.s.)
Screen	Stencil	3–60	0.6–100	30	0.5–60
Inkjet	Digital	0.01–0.5	0.02–5	20	0.001–0.1
Flexo	Polymer Plates	0.17–8	5–180	15	0.01–0.8
Gravure	Engraved Cylinder	0.02–12	8–100	15	0.01–1.1
